# Exploring Links Between Lexical Representations and Cognitive Skills in School-Aged Children with High-Functioning Autism Spectrum Disorder

**DOI:** 10.3390/brainsci15080866

**Published:** 2025-08-14

**Authors:** Vasiliki Zarokanellou, Alexandros Gryparis, Katerina Papanikolaou

**Affiliations:** 1 Department of Speech and Language Therapy, University of Ioannina, 45500 Ioannina, Greece; vzarokanellou@uoi.gr; 2 Department of Child Psychiatry, Medical School, National and Kapodistrian University of Athens, Agia Sophia Children’s Hospital, 11527 Athens, Greece; katpapan@med.uoa.gr

**Keywords:** lexical representations, memory, ASD, school-aged children, vocabulary learning

## Abstract

**Background/Objectives**: The study aimed to investigate how cognitive variables (performance IQ, verbal short-term memory, working memory, and ADHD symptomatology) impact lexical representations in children with high-functioning autism spectrum disorder (HF-ASD). **Methods**: Participants were two groups (n1 = n2 = 20) of monolingual Greek-speaking children, aged 7 to 12 years, with and without HF-ASD matched in age, gender, and cognitive skills. **Results**: Overall, the HF-ASD group had more immature lexical representations than the control group, even though the two groups were similar in naming. In both groups, naming was correlated moderately with verbal short-term memory but only age predicted significantly semantic knowledge. In the ASD group, a bilateral predictive relationship was revealed between output motor programming skills and stored phonological knowledge, supporting theoretical assumptions of the psycholinguistic model of speech. Finally, a different pattern of interrelations was observed between cognitive and lexical variables in the two groups. **Conclusions**: The findings of the current study indicate that ASD children may map and process new vocabulary differently compared to typically developing peers.

## 1. Introduction

Typically developing (TD) children can learn new words remarkably fast and easily after the age of two [1]. Even though there is considerable research on the variables affecting word learning, there is not a broadly accepted theoretical model of word learning describing the factors involved and their inter-relationships. A recent study, applying different latent variables models to investigate the structure of word learning in TD school-aged children suggested two distinct but related phonological and semantic factors [2]. Stackhouse’s and Wells psycholinguistic model [3], which is clinically used to identify underlying speech processing difficulties in children with language and speech disorders proposes that children receive different kinds of information (verbal, visual) about words, which they memorize and store in their mental lexicon in a single underlying representation. This lexical representation gradually becomes more precise including phonological, semantic, motoric, orthographic, and grammatical information. For every known spoken word, three different but linked types of lexical knowledge are stored as follows: phonological representation, semantic representation, and motor program. The semantic representation includes the meaning(s) of the word, while the phonological representation is defined as the sound-based information stored in the mental lexicon for each known lexical item [3]. The phonological representation cannot exist in isolation from semantic representation, since the way a word sounds also determines its meaning(s) [3] (p. 152). For example, without knowing that the round fruit, which typically has thin green or red skin and crisp flesh is labeled apple, a child will be unable to name it upon seeing it in a picture. The motor program encompasses all the necessary articulatory gestures for the pronunciation of a known word and is bilaterally linked with motor programming. Motor programming is a separate component of the model that resembles a storage unit for all legal phonological and phonetic gestural targets, from which one can select those needed to create a new motor program when they have to articulate an unknown word or a non-word. Mature lexical knowledge not only includes accurate underlying representations for all known words but also incorporates the links between these types of representations [3]. 

### 1.1. Word Learning in Children

Word learning proceeds in stages. During the initial fast-mapping phase, children attend to and encode both the phonological form of a new word and its referent, as well as the link between them [2,4]. They then consolidate this information, retaining the new mapping for future use [1,2,4]. Beyond simple retention, children engage in extended-mapping, gradually enriching these initial phonological–semantic associations over time [1,4]. From the above, it becomes clear that several cognitive processes interrelate with vocabulary learning [1,4,5]. To begin with, research has shown that verbal short-term memory (vSTM), verbal working memory (vWM), and visual working memory are linked with vocabulary development in school-aged typical and atypical populations [1,4,5,6,7,8,9,10]. Furthermore, selective attention plays a predominant role in vocabulary learning [11] and in memory encoding and retrieval [5,12,13]. Analytically, exogenous selective attention is needed to follow the gaze of a speaker to an unknown referent [11] and to encode a new phonological representation in vSTM (incidental word learning) [12], while endogenous selective attention and inhibitory control permit the selection of information to be processed and are crucial wherever there is a plethora of stimuli in the environment [12,14]. Thus, information to be learned must be selected, whether the task is implicit or explicit [12,14]. Specifically, in the vocabulary tasks of producing definitions and learning words from context, vWM holds the target word or the reading context in mind while the person needs to search the associated semantic network or infer the meaning of the unknown word, while the endogenous selective attention suppresses irrelevant activated word stimuli, helping the person choose the proper words to describe the lexical item [14,15]. 

### 1.2. Memory Skills in High-Functioning ASD

Most studies agree that children with high-functioning autism spectrum disorder (HF-ASD) present specific memory impairments [16,17,18]. Children with ASD, normal intelligence, and currently normal language ability exhibit soft deficits in episodic memory, leaving procedural and semantic memory intact [17,19,20]. Analytically, these children exhibit difficulties in remembering person-related and emotion-related information [17]. Most studies suggest that children with HF-ASD present significant impairments in working memory (WM) [21,22,23,24], but some researchers disagree [25,26]. WM impairments in ASD are apparent independently of the modality of the task, with deficits in the visual–spatial WM domain being more pronounced than deficits in the vWM [22,24]. Age and IQ are not significant predictors of WM capacity [22,24], but heterogeneous results have been found on the effect of task complexity on WM performance. Some researchers support that the complexity of the material to be stored impacts vWM performance negatively in children with HF-ASD [21,27,28,29]; however, others disagree [22]. Finally, some studies did not find impairments in vWM skills of children with HF-ASD compared to those of TD peers [27,30,31], while others support that children with ASD perform equally or even overperform on semantic or declarative memory tasks compared to their TD peers [32,33,34,35]. To make things worse, the co-existence of Attention Deficit Hyperactivity Disorder (ADHD) may impact the performance of children with ASD in standardized executive function tasks [18,36], possibly affecting memory retrieval and encoding [18]. 

### 1.3. Disparities in Lexical Development in HF-ASD and Possible Links with Memory Abilities

Current research in the field of semantics in individuals with HF-ASD presents conflicting results, as findings support overperformance in standardized naming tests, but deficits in the production of definitions and difficulties in using lexical knowledge appropriately to facilitate performance in free retrieval or organizational tasks [34,35,37,38]. These outcomes suggest that these individuals are capable of learning new verbal materials but have difficulties retrieving them from semantic memory. Findings that give evidence of intact encoding skills and impaired retrieval come from recall tasks. Mottron et al. (2001) [30] reported similar performance in three recall tasks between HF-ASD individuals and controls, as well as similar primacy and recency effects between the two groups. Moreover, the HF-ASD individuals benefited similarly to controls from semantic and phonological cues. These findings imply that people with HF-ASD may have intact encoding abilities. Additionally, Zarokanellou et al. (2023) [37] reported that HF-ASD children produced fewer words in verbal fluency tasks than neurotypical controls, but they did not differ in clustering, switching, and errors in comparison to controls, indicating that the poorer verbal generativity of ASD individuals can be attributed to a slower rate of word retrieval from the semantic network. On the other hand, some children with HF-ASD use words with idiosyncratic meanings and sometimes, they modify the phonological representations of the words or phrases that they produce in an odd-sounding, but comprehensible way. These errors are very similar to those made by younger children when they first learn a new word; however, ASD children retain these errors until later in development and they do not seem to benefit from the corrective feedback of teachers and parents to the same extent as TD peers [34]. Such kinds of language behaviors indicate that HF-ASD children may face problems in encoding new verbal material. Furthermore, children with ASD may use perseverative or pedantic speech, or learn whole sentences without understanding their meaning, revealing that they can learn by rote chunks of language [39]. Additionally, some researchers demonstrated that children with ASD do not use the same encoding strategies as TD peers, grouping together information according to their semantic or categorical characteristics [28,30], while others showed that some children with ASD are more successful than TD peers at mapping phonological forms to novel referents [40], and at false memory or naming tasks [32,35]. Mottron et al. (2001) [30] and Norbury et al. (2010) [40], trying to explain their research’s findings, recommended that word learning and consolidation in ASD children are qualitatively different from those of TD controls and may be driven more by phonological skills, which work compensatory for word learning, counterbalancing the poorer social understanding of ASD children. This different word learning style may be more notable in more demanding tasks such as the production of definitions, which require, to a larger extent, the integration of semantic and phonological features of words.

### 1.4. Purpose 

The current study aimed to investigate the links between lexical representations and cognitive variables (vSTM, visual short-term memory (STM), delayed verbal memory, delayed visual memory, performance IQ, and ADHD symptomatology) in children with HF-ASD to explore possible interrelations and cognitive predictors that may affect vocabulary acquisition in this specific population. Until today research has failed to identify the variable(s) that could explain the significant heterogeneity in lexical semantics skills in children with ASD. A recent study by Sukenik and Tuller (2023) [41] reported that no single factor (age, performance or verbal IQ scores, lexical task type, or linguistic level in other domains) could explain lexical–semantic ability in children with ASD although children with more severe language impairment generally, but not globally, also presented deficits in vocabulary.

### 1.5. Research Questions

1. Is there an association between cognitive skills (performance IQ, immediate and delayed vSTM, immediate and delayed visuospatial memory, and ADHD symptomatology) and lexical representations (semantic representation, phonological representation, and motor program/motor programming) in children with HF-ASD? 

2. How does that pattern of interrelations between cognitive skills and lexical representations differ between children with HF-ASD and TD peers? 

3. To what extent can cognitive skills predict the lexical representations of school-aged children with ASD and vice versa? 

## 2. Materials and Methods

### 2.1. Participants

Twenty children with HF-ASD (16 boys, 4 girls), aged 6 years and 8 months to 12 years old (Mean: 9 years and 11 months, *SD* = 18.1 months), and twenty TD children (15 boys, 5 girls), aged 6;4 to 11;11 years old (Mean: 9 years and 10 months, *SD* = 17.3 months) participated. All participants were monolingual Greek-speaking children who attended a regular elementary school, each with a Performance and Verbal IQ ≥ 70 according to Raven’s Educational [42]. All participants with HF-ASD had been recruited from an ASD clinic of a University Child Adolescence Psychiatric Department at a Children’s Hospital. They have been given a formal diagnosis of ASD without accompanying intellectual impairment (Level 1-Requiring support) according to DSM-5 criteria [43] by an experienced child psychiatrist in the diagnosis of autism. Children in the control group were recruited from two different regular elementary schools and were TD as understood from their educational records, and teacher and parents’ reports. All TD participants had a score of under 11 in the Greek edition of the Social Communication Questionnaire [44] and 93% on the Greek Assessment Scale for ADHD-IV [45] as completed by their parents. The two groups were similar in age, gender, PIQ, and memory skills (Table 1). 

### 2.2. Procedure

The study was approved by the Ethics Committee of the Greek Institution of Educational Policy (Φ15/176437/204757/Δ1) and the Ethics Committee of the Medical School of Athens. Before the individual assessment of the participants, all caregivers provided written consent and completed the Greek version of the Social Communication Questionnaire (SCQ, Lifetime Form) [44], as well as the Greek Assessment Scale for Attention Deficit Hyperactivity Disorder-IV [45]. For the cognitive assessment of the participants, the Greek edition of Raven’s Educational [42] was administered, followed by the Memory Assessment Battery for preschool and school-aged children [46]. Two vocabulary tests were given in a counterbalanced order to assess vocabulary knowledge: a naming test (the Greek version of Expressive One Word Picture Vocabulary Test-Revised, (EOWPVT-R0, [47], which assesses lexical breadth, and a word definition test (Crichton Vocabulary Scale), which examines lexical depth. Phonological representations were evaluated with the administration of two different tasks, specifically a single-word naming task and a repetition of non-words task. Participants’ answers were audio-recorded with a digital voice recorder (model OLYMPUS WS-831) and subsequently transferred to their corresponding answer sheets.

### 2.3. Screening and Cognitive Assessment

To exclude children with neurodevelopmental disorders from the control group and to match participants for cognitive skills, the following tools were administered:

#### 2.3.1. The Greek Version of the Social Communication Questionnaire (SCQ, Lifetime Form) [44]

This questionnaire is a standardized screening instrument for ASD symptomatology in individuals over 4 years old with cognitive functioning above the age of two. The Lifetime form of the questionnaire refers to the whole developmental history of each participant and is easily completed by the child’s primary caregiver in less than 10 min. In this study, the SCQ served both to screen out potential ASD cases from the control group and to assess ASD severity within the clinical sample. Pilot standardization demonstrated good internal consistency, with Cronbach’s α exceeding 0.70 for each subscale and reaching 0.91 for the total score. A threshold of ≥11 was identified as optimal for distinguishing HF-ASD from non-spectrum participants, yielding 90.6% sensitivity and 89.6% specificity. By comparison, another Greek validation of the same tool [48] recommended a cut-off of ≥12, which achieved 88.7% sensitivity and 90.6% specificity. We therefore adopted 11 as our study’s SCQ cut-off. 

#### 2.3.2. The Greek Assessment Scale for Attention Deficit Hyperactivity Disorder-IV (Parent Form) [45]

The parent form of the Greek-standardized ADHD Rating Scale–IV (DuPaul et al. 1998) was used to screen for ADHD symptoms. This 18-item checklist, based on DSM-IV criteria, employs a 4-point Likert scale (0 = almost never to 4 = often) and provides normative data for children and adolescents aged 5 to 19 years. The scale demonstrates strong internal consistency (Cronbach’s α = 0.89). Odd-numbered items assess inattention; even-numbered items assess hyperactivity. Raw scores were converted to percentiles, and a cut-off at the 93rd percentile—recommended by the authors—was used to identify probable ADHD (sensitivity = 74%, specificity = 70%, PPV = 72%, NPV = 72%). This threshold served both to exclude potential ADHD cases from the control group and to quantify ADHD symptomatology in the HF-ASD group. 

#### 2.3.3. The Raven’s Educational [42]

Raven’s Educational assesses intelligence and it is standardized for monolingual Greek-speaking children, aged 4 to 12 years old. It consists of two scales: the Raven’s Colored Progressive Matrices (CPM), which assess performance intelligence (PIQ), and the Crichton Vocabulary Scales (CVS), which assess verbal intelligence (Verbal IQ) through the task of the production of definitions. Both scales present high reliability and internal consistency. Specifically, the standardization of CPM revealed a total Cronbach’s alpha coefficient equal to 0.903 and a Guttmen split-half coefficient equal to 0.832, while the CVS scale showed a total Cronbach’s alpha coefficient equal to 0.986 and a Guttmen split-half coefficient equal to 0.967. In the present study, Raven’s Educational was administered according to the manual guidelines to exclude participants with PIQ or VIQ < 70 and to match study groups in PIQ. For the CPM test the total raw score range is between 0 and 36 points, while for the CVS, the total raw score range varies between 0 and 160 points. Raw scores can be converted to standard scores and percentiles. 

#### 2.3.4. The Memory Assessment Battery for Preschool and School-Aged Children [46]

The Memory Assessment Battery [46] is a standardized measurement that can identify and assess possible memory deficits in monolingual Greek-speaking children, aged 5 to 12 years old. The tool includes (a) a screening assessment test for verbal and visual STM, (b) a retrieval of stories subtest, and (c) a retrieval of visual information subtest. The battery assesses immediate and delayed memory. All memory scales were implemented, and the scoring of each subtest was performed according to the guidelines of the manual. In the current study we used raw scores for the comparison of the participant’s performance. According to the test’s manual, raw scores can be converted to standard scores and percentiles. A short description of each scale is provided below:

For the assessment of vSTM, the child had to repeat a list of five or seven unrelated words in five attempts. The examiner read the list of words in a steady rhythm to the child and asked him/her to repeat it. For children aged 5to 8 years and 11 months, the list included five words, while for older children (9 to 12), it contained seven. The score was calculated according to the attempts the child needed to recall all lexical items and the words they remembered in each attempt. Delayed verbal memory was evaluated by asking the child to repeat the same list of words after a short break of five to ten minutes. 

For the assessment of visual STM, each examinee had to put back five or seven chips in the correct position in a frame, presenting 12 white squares, reproducing the pattern the examiner had made. For children aged 5 to 8 years and 11 months, there were five chips, while for older children there were seven. Each child had a total of five trials and before every attempt, the examinee showed the correct pattern to the child. The score for this task was calculated similarly to the vSTM task. Delayed visual memory was assessed by asking the child to reproduce the same pattern with chips after a short break of five to ten minutes. 

For the assessment of narrative memory, the examiner read two short stories to each examinee and asked the examinee to retell each story after they had heard it. The performance in this subtest was calculated based on the important information the child recalled from each story. For the assessment of delayed narrative memory, the examiner asked the child to narrate each story again, after a short break of 20 to 30 min. 

Finally, the assessment of visual information was similar to that of visual STM, except the 12-squared frame included the images of two suitcases and two baskets, and the chips had two different colors (red and yellow). Again, the younger children (5 to 8 years and 11 months) had to put five chips in the correct position, and the older children (9 to 12 years) had to place seven, reproducing the design of the examiner. Again, each child had five attempts in total, and the score was calculated according to the number of chips with the right color the examinee placed in the right position in each trial, and the number of trials it took them to achieve the goal. Delayed visual information recall was assessed by asking the child to reproduce the same pattern with chips after a short break of 20 to 30 min. In all statistical analysis, the scoring from the visual information subtest was used. 

### 2.4. Lexical Representations Assessment

To assess the lexical representations of participants, the following measures were implemented:

#### 2.4.1. Semantic Representations

For the assessment of semantic representations, the Greek version of the EOWPVT-R [47] and the CVS [42] were given. The Greek version of the EOWPVT-R [47] consists of 100 words presented through line drawings and evaluates naming ability (vocabulary breadth) in children 2 to 12 years old. The target words are mainly high-frequency and low-frequency nouns and a few verbs. In the present study, a translated and adapted version of this tool was given, which has been used in previous Greek studies [37,38]. The administration of the test began at the beginning, regardless of the participant’s age, and stopped when the child made 6 consecutive mistakes. Every correct answer is awarded one point whereas wrong answers receive zero points, leading to a total raw score range between 0 and 100. Correct answers in the naming task were considered all responses that approximated the adult-like production of the target stimulus, even if they included articulatory errors. The CVS [42] evaluates vocabulary depth by asking children to define words. Standardized for monolingual Greek-speaking 4- to 12-year-olds, it comprises two lists of 40 words each. The examiner names each word and prompts the child to explain its meaning; vague definitions can be probed once, but after four consecutive incorrect responses on a list, administration moves on (or ends, if it is the second list). Scoring allocates 2 points for definitions that include a correct semantic category, synonym, metaphorical meaning, or two key features; 1 point for partial or less critical semantic details; and 0 for incorrect or irrelevant answers. Total raw scores range from 0 to 160 and can be converted into standard scores and percentiles.

#### 2.4.2. Phonological Representations

To evaluate the accuracy of phonological representations of HF-ASD children, we implemented two different phonological tasks, namely a naming task and a non-word repetition task according to best practice’s recommendations for administering both tasks of imitated and spontaneous speech in speech assessment [49]. In the naming task, children have to recall and access a stored phonological representation and its related motor program from the long-term memory, whereas in non-word repetition, prior lexical knowledge cannot be accessed. Non-word repetition closely reflects the speech processing a child makes when they learn a novel word and it has been used commonly to assess a child’s ability to create new motor programs, thus evaluating the functioning of motor programming [3] (p. 163). Specifically, the precise imitation of a non-word includes many skills such as speech perception, encoding or segmenting the speech sounds of non-word stimulus, storing the new phonological representation in memory, accessing this representation, planning the motor action required to reproduce the speech sounds of the stimulus, and proceeding to the execution of those articulatory movements [50].

In the naming task, we used the first 35 target words of the standardized Greek Test for Phonological and Phonetic Development [51]. In Greek, the phonological and phonetic development is almost perfectly completed by the age of six and standardized norms exist for this age range [51,52]. Participant’s productions were cross-examined in relationship to neurotypical controls for the total number of articulation errors, whereas two different phonological indices, namely the Whole Word Match (WWM) index [53] and the Percentage of Consonant Correct (PCC) index [54] were also calculated. The WWM and PCC indices are global measurements, which are used as measures of the accuracy of phonological representations. The WWM shows the yes–no congruence of adult and child productions of a word [53], while the PCC index measures the number of correctly articulated consonant sounds in relation to the total number of consonants sounds included in the target stimuli [54]. The WWM index in comparison to PCC index assesses phonological development taking also into account the phonological properties of word stimuli such as word length, word phonotactic structure, and stress, thus enhancing content validity. All answers of participants were recorded and phonologically transcribed using the International Phonetic Alphabet in the proper record form. Small distortions of narrow phonetic transcription such as partial devoicing, frontal lisping, etc., were ignored throughout the analysis. Each correct answer based on the adult production received one point, and its misarticulated response zero, leading to a total raw score between 0 and 35 for the WWM index. 

The non-word repetition scale [55] is a standardized scale, which consists of 24 non-words, which gradually become more complex in structure, resembling disyllabic, trisyllabic, four syllable and five syllable real Greek words in structure (e.g., /’iva/,/apir’cis/,/di’vastelo/,/eloma’tiro/) [55]. In Greek, most words are disyllabic or multisyllabic (3–5 syllables), and the syllable structures V, CV, CVC, CCV, CCVC, and VC are the predominant ones [56]. Previous studies have shown that performance accuracy on non-word repetition tasks is influenced by the phonotactic probability of the non-word stimuli implemented. Moreover, researchers suggest that non-word repetition tasks using multisyllabic stimuli evaluates the quality of phonological representations, since poor non-word repetition may indicate an impairment in the integration of a phonological stimulus into a cognitive form that is easily accessible for speech production [57,58]. During task administration, the examiner covers his mouth with one hand and reads each non-word only once, while the child must repeat the non-word afterwards. Repetition is scored as incorrect if a speech error of any type is made. Minor speech distortions of narrow phonetic transcription were ignored. After three consecutive inaccurate productions, the administration of the test stops. Each accurate production is awarded one point, while every incorrect production takes zero points, leading to a total raw score range from 0 to 24. The raw scores of the scale can be converted to standard scores. This scale is the only standardized measure for non-words in Greek and is standardized for children in the first and second grade of elementary school. In the current study for the comparison between groups, raw scores were used. 

In summary, the tasks and measurements used to assess lexical representations are presented in Table 2: 

### 2.5. Data Analysis

Quantitative variables were expressed as mean (standard deviation), while qualitative variables were expressed as absolute and relative (%) frequencies. All quantitative variables were assessed for normality using the Shapiro–Wilk test, complemented by visual inspection of Q–Q plots and histograms. For the comparison of proportions, chi-square, and Fisher’s exact tests were used. Student’s *t*-test was used for the comparison of continuous variables between the two groups. Spearman correlation coefficient (rho) was implemented to examine the correlations between the cognitive and lexical variables in each group separately. Furthermore, to investigate the impact of various factors on specific outcome variables, multiple regression analyses were performed separately for each dependent variable. All regression assumptions (e.g., normality of residuals, linearity, etc.) were checked. Since this is an exploratory study, no sample size estimation was performed. All reported *p*-values are two-tailed. Statistical significance was set at *p* < 0.05 and analyses were conducted using IBM SPSS statistical software (version 29.0) [59].

## 3. Results 

### 3.1. Lexical Skills of Participants 

Overall, ASD participants performed significantly lower in the production of definitions task (*p* = 0.045; Cohen’s *d*: −0.650), but not in naming skills (*p* = 0.761; Cohen’s *d:* 0.100) in comparison to their TD peers. Moreover, the ASD group made significantly more phonological errors (*p* = 0.004; Cohen’s *d*: −0.687) and had significantly lower performance in the WWM index (*p* = 0.003; Cohen’s *d*: −0.663) and the PPC index (*p* = 0.004, Cohen’s *d* = −0.687) in comparison to their control group (Table 3). 

### 3.2. Relationships Between Lexical and Cognitive Variables in TD and ASD Groups

#### 3.2.1. TD Group

Table 4 presents the correlations between the cognitive and the lexical variables in the TD group. 

There are significant and strong associations between the different types of memory. Analytically, there was a moderate correlation between vSTM and delayed verbal memory (*r* = 0.478; *p* = 0.033), visual memory and delayed visual memory (*r* = 0.496; *p* = 0.026), delayed visual memory and vSTM (*r* = 0.479; *p* = 0.033), delayed verbal memory and visual memory (*r* = 0.482; *p* = 0.031) and delayed visual and verbal memory (*r* = 0.699; *p* = 0.001). The PIQ score does not correlate significantly with any cognitive or lexical variable, while the verbal IQ score (CVS) shows moderate negative and significant associations with delayed verbal memory (*r* = −0.575, *p* = 0.008) and visual memory (*r* = −0.604; *p* = 0.005). Furthermore, ADHD scores present a moderate and significant association with vSTM (*r* = 0.461; *p* = 0.041), while naming skills (EOWPVT-R) correlate significantly and moderately to highly with vSTM (*r* = 0.689; *p* = 0.001). Finally, the PCC index and the WWM index correlate perfectly but since the participants of the control group had acquired the adult model of speech, this perfect association is expected (*r* = 1.000; *p* < 0.0001).

#### 3.2.2. ASD Group

Table 5 presents the correlations between the cognitive and the lexical variables in the ASD group. 

There were strong and significant associations between the different types of memory. Analytically, there were strong or moderate correlations between vSTM and delayed verbal memory (*r* = 0.751; *p* = 0.000), visual memory and delayed visual memory (*r* = 0.774; *p* = 0.000), delayed visual memory and vSTM (*r* = 0.684; *p* = 0.001), delayed verbal memory and visual memory (*r* = 0.483; *p* = 0.031) and delayed visual and verbal memory (*r* = 0.496; *p* = 0.026). Contrary to the control group, in the ASD group there was a moderate and significant association between vSTM and visual memory (*r* = 0.675; *p* = 0.001). Moreover, the vSTM showed positive medium associations with naming skills, as measured by the EOWPVT-R (*r* = 0.457; *p* = 0.043), and phonological performance, as measured by the indices PCC (*r* = 0.514; *p* = 0.021) and WWM (*r* = 0.510; *p* = 0.022). In the ASD group, the performance and verbal IQ scores did not correlate significantly with any other cognitive or lexical variable. Furthermore, ADHD scores were associated negatively and significantly with the repetition of non-words (*r* = −0.453; *p* = 0.045) but with no other cognitive or lexical variable, while repetition of non-words were moderately correlated with the two phonological indices used, namely PCC (*r* = 0.595; *p* = 0.006) and WWM (*r* = 0.597; *p* = 0.005) and naming skills (*r* = 0.484; *p* = 0.031). Finally, the PCC and WWM indices were very highly, positively, and significantly correlated with each other (*r* = 0.999; *p* = 0.000).

### 3.3. Relationships Between Predictors and Lexical Representations in TD and ASD Groups

To further explore to what extent age, vSTM, and severity of ADHD affect lexical development, classical multiple regression analyses were conducted. Multiple regression analysis was performed independently for each group. We used only the cognitive and lexical skills that were significantly correlated in each group as dependent and independent variables. Age was selected as an independent factor in all multiple regression analyses in both groups (ASD and TD) as lexical knowledge improves with age.

In the TD group, two different multiple regression analyses were performed to assess the impact of age and memory capacity on semantic knowledge (Table 6). In the first regression, we examined the effect of age, visual memory, and delayed visual memory on the production of definitions ability. Regression results revealed that only age weakly and significantly predicted the production of definition performance, explaining 20.8% of the variance in the production of definition skills (*R*^2^ = 0.333, F (3,16) = 2.662, *p* = 0.083). Similarly, naming skills were predicted moderately by age, which explained 47.8% of the variance of this lexical variable (*R*^2^ = 0.533, F (2,17) = 9.691, *p* = 0.002). In this group, since there were no other significant correlations between cognitive and lexical variables, no other regression analysis was conducted. 

Finally, to explore the impact of expressive vocabulary on vSTM, a separate multiple regression was conducted (Table 7). For this group of participants, none of the measures used, namely age, ADHD scores, and naming skills significantly predicted vSTM performance.

We performed four multiple regression analyses for the ASD group with dependent variables naming scores, the PCC and WWM indices, and non-word repetition scores (one at a time) (Table 8). To investigate the impact of age, vSTM, and repetition of non-word ability on naming skills or PCC/WWM index (dependent variable), three multiple regression analyses were performed separately for each dependent variable. Results indicated that only age was a significant predictor of naming skills in the ASD group, explaining 47.7% of the variance in naming performance (*R*^2^ = 0.56, F (3,16) = 6.782, *p* = 0.004). Additionally, regression results revealed that only repetition of non-words significantly predicted the number of articulatory errors as measured by the PCC index, explaining 59.8% of the variance in PCC index (*R*^2^ = 0.661, F (3,16) = 10.405, *p* < 0.001), and the accuracy of phonological representations based on an adult model of speech production, explaining 54.2% of the variance in WWM index (*R*^2^ = 0.615, F (3,16) = 8.506, *p* = 0.001), respectively. Moreover, to investigate the impact of age, ADHD symptomatology, naming skills, and articulatory ability as measured by the PCC index on repetition of non-words, another multiple regression was performed. Results showed that only the PCC index significantly and strongly predicted repetition of non-words explaining 64.2% of the variance in non-word repetition scores (*R*^2^ = 0.717, F (4,15) = 9.521, *p* < 0.001). 

Finally, to investigate the impact of lexical knowledge on vSTM we fit separate multiple regressions. We used two different multiple regression models to examine the influence of age, naming skills, and phonological skills as measured by the PCC index or the WWM index on vSTM performance (dependent variable) (Table 9). Regression results returned that only age significantly and weakly predicted vSTM capacity, explaining 29.4% or 28.7% of the variance of vSTM performance, respectively. All regression assumptions were satisfactorily met in all analyses.

## 4. Discussion

The present study examined lexical representations in school-aged children with typical development (TD) and high-functioning autism spectrum disorder (HF-ASD) using Stackhouse and Wells’s (1997) model [3]. We sought to clarify how these representations relate to cognitive abilities—PIQ, memory skills, and ADHD symptomatology—and to identify which factors might account for the wide variability in lexical performance seen among children with ASD. To do so, we explored how the pattern of interrelations between cognitive variables and lexical representations differed between children with HF-ASD and a group of cognitively matched TD peers. Finally, we wanted to explore the role of ADHD, vSTM, and visual memory in lexical acquisition in children with HF-ASD. More specifically, we evaluated which variables (age, vSTM, visual memory, ADHD symptomatology) could predict each type of lexical representation and if lexical knowledge could predict vSTM. Despite extensive research, no prior study has pinpointed the variables that explain heterogeneity in lexical semantics among children with ASD [41]. The results of the study revealed weaker lexical representations in the HF-ASD participants in comparison to controls, age-related semantic learning in both groups and atypical cognitive-lexical associations in the HF-ASD group, which support the notion of divergent lexical acquisition in HF-ASD individuals. An analytical discussion of the study’s findings follows:

### 4.1. Lexical Representations in Children with HF-ASD

Our results show that children with HF-ASD exhibit weaker stored linguistic information—particularly less accurate phonological representations and shallower lexical depth, as evidenced by their lower performance on the definition-production task. In contrast, the HF-ASD group’s scores on all memory scales (verbal and visual STM, plus rehearsal-based secondary memory) matched those of controls, indicating that their deficits are confined to language rather than memory. Production of definitions constitute a measure of the concreteness and precision with which a word is represented in the mental lexicon. Consistent with prior research [34,38,40], our participants with HF-ASD excelled at naming but struggled to define words accurately and sometimes produced misarticulations.

### 4.2. Relationships Between Lexical and Cognitive Variables

Regarding our first research question whether there are correlations between the measures of lexical representations and the measures of cognitive skills, Spearman’s analysis revealed significant correlations between the different types of lexical knowledge (lexical representations) and cognitive variables. Additionally, the statistical analysis showed significant moderate correlations: a. between the different memory tasks in the two groups of participants, supporting the structure of Baddeley’s model of working memory, and b. between stored lexical knowledge and output motor programming skills in the ASD group as the psycholinguistic model of Stackhouse and Well’s (1997) [3] suggests. The PIQ score was not correlated with any other cognitive or lexical variable in both groups of participants, a finding which can be explained by the fact that both samples included children with a typical IQ range.

Baddeley’s model (2000) [7] includes two domain-specific storage devices (phonological loop/visuospatial sketchpad) that retain verbal or visual information for a few seconds before they fade, and two more general storage devices namely, the central executive and the episodic buffer which bind information from the two domain-specific systems and coordinate them and their interactions with long-term memory since they act as subsidiary systems between short-term and long-term memory. This dynamic interaction between domain-specific storage components (phonological loop/visuospatial sketchpad) and domain-general storage components allows WM the capacity to address the role of active memory in real-world cognitive situations. In the control group, the statistical analysis showed moderate correlations between vSTM and delayed verbal memory, visual STM and delayed visual memory, delayed visual memory and vSTM, delayed verbal memory and visual STM, and delayed visual and verbal memory. However, there was not a significant correlation between verbal and visual STM tasks. The above results support the interactions between the different memory subsystems Baddeley’s model describes and they reinforce the domain-specific roles of phonological loop and visuospatial sketchpad devices. Similar or higher correlations between different memory measures were also observed in the ASD group, but contrary to the control group, the ASD group presented a significant moderate correlation between the vSTM task and the visual STM task. This finding may indicate that children with ASD present enhanced visual functioning, storing language units, in our case, words as images, as relevant research proposes [60]. 

Regarding semantic representations, in both groups, naming capacity was correlated significantly and moderately with vSTM, underpinning findings from other studies in typical and atypical populations [6,7,8], whereas the production of definition task, presented a moderate negative correlation with delayed verbal memory and visual STM, only in the control group.

Moreover, in the control group, the ADHD scores showed a moderate significant association with vSTM performance, while in the ASD group, they presented a moderate negative association with the repetition of non-word scores. As stated above, the non-word repetition task is a measurement that has been used broadly in research on children with language and speech impairments, either as a measure of motor programming ability, since it does not allow the child to access stored lexical knowledge in long-term memory to facilitate articulatory performance, or as the phonological loop’s capacity of storage, since it resembles the process a child undertakes to create a new phonological representation for an unknown word [3,9]. Taking together the above, our results underscore the important effect of attention in the storage of verbal information, a finding that is extensively supported by relevant research [5,11,12,13]. 

Finally, in the ASD group, the performance on the non-word repetition task was significantly and moderately associated with naming skills and the two phonological indices (PCC/WWM). These findings are noticeable since they corroborate Stackhouse and Well’s theoretical model [3]. 

To summarize, our analysis revealed that children with HF-ASD and their TD peers display different patterns of association between cognitive and lexical measures. In the ASD group, verbal and visual STM performance were strongly correlated, suggesting a distinct strategy for encoding new information within domain-specific working memory systems. This finding aligns with clinical observations that many individuals on the autism spectrum rely on visual learning, and with meta-analytic evidence of atypical engagement of visual processing during written-word recognition, all of which support the idea that children with ASD may encode verbal material as mental images [60]. Moreover, only the ASD group showed moderate correlations between non-word repetition and both naming and phonological accuracy (PCC and WWM indices), despite performing similarly to controls on these tasks. These associations underscore a unique profile of speech processing in HF-ASD and lend support to the Stackhouse and Wells (1997) psycholinguistic model [3], which posits oral–motor output skills (motor program and motor programming) and stored phonological and semantic representations are closely linked.

### 4.3. Relationships Between Predictors and Lexical Representations 

To examine how age, memory abilities, and ADHD symptoms influence lexical knowledge in our school-aged samples, we conducted classical regression analyses. In both groups, only age significantly predicted semantic knowledge: it accounted for 47.8% of variance in naming ability and 20.8% in definition performance among controls, mirroring well-documented age-related vocabulary growth in typical development [34], and explained 47.7% of naming variance in the ASD group. These results contradict the findings of Sukenik and Tuller’s (2023) study [41], which proposed that no single factor (age, type of vocabulary task, IQ score, language skills) could explain lexical–semantic knowledge in children with ASD. For the control group, no other regression analysis was performed since there were not other significant correlations between other lexical and cognitive variables. In the ASD cohort, non-word repetition emerged as the sole significant predictor of phonological accuracy—explaining 54.2% of variance in the WWM index—and also accounted for 59.8% of variance in PCC scores. Conversely, only PCC predicted non-word repetition performance (64.2% of variance), underscoring a reciprocal relationship between output motor programming and stored phonological knowledge. Summarizing the above, the findings support the notion that accurate phonological representations make it easier to reproduce legal non-words, which follow the phonotactic rules of the mother language and provide evidence for a bilateral predictive relationship between output motor programming skills and stored phonological knowledge, supporting the theoretical assumptions of the psycholinguistic model of speech processing. 

Finally, in the TD group, none of our predictors accounted for variance in verbal STM, reflecting the well-documented individual differences in vSTM capacity among typically developing children even of the same age [6]. In contrast, age emerged as a weak but significant predictor of vSTM in the HF-ASD group, explaining approximately 28–29% of its variance. Research findings also note significant individual differences in vocabulary performance in TD children of the same age range, whereas they provide evidence for a significant association between vSTM and vocabulary, an association which was also found in our study, even though naming capacity was not found to be a predictive variable of vSTM skills [6].

## 5. Conclusions

To the best of our knowledge, this study is among the few to assess cross-sectional lexical knowledge alongside memory skills in school-aged HF-ASD children. The findings indicate that lexical disparities in children with ASD may not result from deficits in memory skills but rather reflect a distinctive learning style. Since vocabulary development constitutes the cornerstone of language development, it is vitally important to define possible risk factors that could jeopardize lexical acquisition in this specific population. Contrary, to other research [41], our findings revealed that age was a significant predictor of naming performance in the HF-ASD group. Furthermore, the repetition of non-words was a significant predictor of the accuracy of phonological representations and there was a bilateral association between non-word repetition and stored phonological and semantic knowledge in the ASD group. Moreover, the ADHD scores were significantly and negatively correlated with the repetition of non-word scores, implying that ADHD symptoms may negatively affect the fast-mapping stage of word learning in HF-ASD children. 

## 6. Limitations and Future Directions

The present study is an exploratory study, examining the interplay between lexical representations and cognitive variables (e.g., immediate and delayed memory, PIQ, ADHD symptomatology) in school-aged children with HF-ASD. We found that, in the ASD group, higher ADHD symptomatology was associated with poorer non-word repetition, highlighting comorbid ADHD as a potential risk factor for lexical disparities that have received limited attention. However, our small sample and the absence of an ASD-only (no ADHD) comparison group represent key limitations. Future research should recruit larger, more diverse cohorts—including ASD without ADHD, ASD with ADHD, and typically developing controls—to confirm these findings and clarify the unique contributions of ADHD symptoms to lexical acquisition in HF-ASD.

## Figures and Tables

**Table 1:** Participant’s characteristics.

	Group		
	TD Group	ASD Group	*p*-Value
	Mean (*SD*)	Mean (*SD*)	
Age (months)	117.7 (17.3)	119.3 (18.1)	0.776
Gender, Ν (%)			
Boys	15 (75.0)	16 (80.0)	0.705
Girls	5 (25.0)	4 (20.0)	
Total Score SCQ	5.2 (2.5)	13.9 (8.1)	<0.001
Total Score ADHD	8.4 (5.1)	21.5 (12.7)	0.002
Raven’s Educational			
Raven’s Coloured Matrices (PIQ)	108.8 (15.8)	109.5 (14.1)	0.902
PIQ range	80–135	90–135	-
Crichton Vocabulary Scale (VIQ)	96.5 (12.2)	88.5 (12.3)	0.027
VIQ range	80–130	80–120	-
Memory Assessment Battery (raw scores)			
vSTM	27.3 (4.6)	28.7 (5.9)	0.166
Delayed vSTM	6.1 (1.1)	5.9 (1.9)	0.166
Narrative Memory	47.1 (11.6)	49.5 (13.5)	0.507
Delayed Narrative Memory	43.9 (12.3)	41.1 (17.6)	0.645
Visual Information	64 (8.6)	62.4 (11.4)	0.854
Delayed visuospatial memory	12.5 (2.0)	11.5 (3.1)	0.284

**Table 2:** Tools and measures used for assessing lexical representations.

Lexical Representations	Task	Tool	What It Measures	Unit of Measurement
Semantic representation	Naming task	Expressive one Word Picture Vocabulary Test [47]	The ability to recall the phonological representation of a word from the long-term memory, using a picture stimulus	Total raw score
	Production of definition task	Crichton Vocabulary Scale [42]	The ability to accurately define a word presenting the most significant semantic features of the spoken word stimulus given	Standard score
Phonological Representation	A single-naming task of 35 words	Test of Phonetic and Phonological Development [51]	The phonological precision of the produced word stimulus	Total raw scores of articulation errors, WWM index, PCC index
Motor programming	Non-word repetition	Non-word repetition scale [55]	The ability to create a new motor program	Total raw score of the correct productions of non-words

**Table 3:** Lexical skills of participants.

	Group			
	TD Group (n1 = 20)	ASD Group (n2 = 20)	*p*-Value	Cohen’s *d*
	Mean (*SD*)	Mean (*SD*)		
Semantic Representation				
EOWPVT-R (RS)	69.90 (9.6)	69.00 (9.0)	0.761	0.100
CVS (VIQ)	96.50 (12.2)	88.50 (12.3)	0.045	−0.650
Phonological Representation				
Phonological errors (RS)	0.20 (0.70)	3.0 (5.70)	0.004	−0.688
WWM Index	1.000 (0.002)	0.985(0.031)	0.003	−0.663
PCC Index	0.999 (0.007)	0.974 (0.05)	0.004	−0.687
Repetition of non-words (RS)	20.5 (2.2)	17.6 (6.2)	0.261	0.622

RS = raw score.

**Table 4:** Spearman’s correlations between cognitive and lexical variables in the TD group.

	1	2	3	4	5	6	7	8	9	10
(1) RS ADHD										
(2) PIQ	0.028									
(3) vSTM	0.461 *	0.098								
(4) Delayed vSTM	0.145	−0.162	0.478 *							
(5) visual Information	−0.082	−0.020	0.147	0.482 *						
(6) Delayed Visual Information	0.394	−0.212	0.479 *	0.699 **	0.496 *					
(7) VIQ	0.248	0.173	−0.001	−0.575 **	−0.604 **	−0.421				
(8) RS EOWPVT-R	0.395	0.229	0.689 **	0.406	0.149	0.350	0.027			
(9) PCC index	0.080	0.263	0.200	0.254	0.286	0.286	−0.244	0.380		
(10) WWM Index	0.080	0.263	0.200	0.254	0.286	0.286	−0.244	0.380	1.000 **	
(11) RS non-words	−0.163	0.067	−0.141	−0.289	0.216	−0.207	−0.299	−0.088	0.305	0.305

*. Correlation is significant at the 0.05 level (two-tailed), RS = raw score; PIQ = Performance IQ; VIQ = Verbal IQ; PCC = Percentage of Consonant Correct; WWM = Whole Word Match; **. Correlation is significant at the 0.01 level (two-tailed).

**Table 5:** Spearman’s correlations between cognitive and lexical variables in the ASD group.

	1	2	3	4	5	6	7	8	9	10
(1) RS ADHD										
(2) PIQ	−0.242									
(3) vSTM	−0.111	−0.175								
(4) Delayed vSTM	−0.004	0.032	0.751 **							
(5) Visual information	0.004	−0.285	0.675 **	0.483 *						
(6) Delayed visual information	−0.397	−0.130	0.684 **	0.496 *	0.774 **					
(7) VIQ	0.156	0.237	0.173	0.191	0.341	0.237				
(8) RS EOWPVT-R	−0.428	−0.048	0.457 *	0.295	0.071	0.245	−0.211			
(9) PCC index	0.025	−0.243	0.514 *	0.276	0.313	0.192	−0.047	0.400		
(10) WWM index	0.027	−0.246	0.510 *	0.276	0.300	0.179	−0.065	0.406	0.999 **	
(11) RS non-words	−0.453 *	0.096	0.237	0.187	0.129	0.223	−0.149	0.484 *	0.595 **	0.597 **

*. Correlation is significant at the 0.05 level (two-tailed), RS = raw score; PIQ = Performance IQ; VIQ = Verbal IQ; PCC = Percentage of Consonant Correct; WWM = Whole Word Match; **. Correlation is significant at the 0.01 level (two-tailed).

**Table 6:** Multiple regression analyses for the TD group predicting lexical skills.

Dependent Variables	Independent Variables	B	SE	t	*p*	*R* ^2^
Production of definitions	Age	−0.372	0.173	−2.155	0.047	
	Visual STM	0.407	0.415	0.980	0.342	
	Delayed visual memory	1.161	1.653	0.702	0.493	
Full model		94.231	20.696	4.553	<0.001	0.333
Naming (EOWPVT-R)	Age	0.312	0.102	3.074	0.007	
	vSTM	0.576	0.379	1.522	0.146	
Full model		17.427	12.072	1.444	0.167	0.533

**Table 7:** Multiple analysis for the TD predicting vSTM.

Dependent Variables	Independent Variables	B	SE	t	*p*	*R* ^2^
vSTM	Age	0.066	0.075	0.881	0.392	
	ADHD	0.304	0.193	1.573	0.135	
	Naming (EOWPVT-R)	0.122	0.142	0.857	0.404	
Full model		8.486	7.109	1.194	0.250	0.379

**Table 8:** Multiple regression analyses for the ASD group predicting lexical skills.

Dependent Variables	Independent Variables	B	SE	t	*p*	*R* ^2^
Naming	Age	0.371	0.105	3.521	0.003	
	vSTM	−0.350	0.336	−1.042	0.313	
	Non-words	0.479	0.0258	1.859	0.081	
Full model		26.273	10.215	2.572	0.020	0.560
PCC index	Age	0.000	0.001	0.878	0.393	
	vSTM	−0.002	0.002	−1.028	0.319	
	Non-words	0.007	0.001	5.339	<0.001	
Full model		0.850	0.050	17.101	<0.001	0.661
WWM index	Age	0.000	0.000	1.222	0.239	
	vSTM	−0.001	0.001	−1.125	0.277	
	Non-words	0.004	0.001	4.726	<0.001	
Full model		0.902	0.033	27.537	<0.001	0.615
Non-words	Age	−0.013	0.064	−0.203	0.842	
	ADHD	−0.145	0.074	−1.955	0.069	
	Naming	−0.009	0.146	−0.059	0.954	
	PCC index	100.410	19.119	5.252	<0.001	
Full model		−74.926	16.995	−4.409	<0.001	0.717

**Table 9:** Multiple analysis for the ASD predicting vSTM.

Dependent Variables	Independent Variables	B	SE	t	*p*	*R* ^2^
vSTM	Age	0.236	0.086	2.732	0.015	
	Naming (EOWPVT-R)	−0.119	0.185	−0.644	0.529	
	PCC index	13.837	25.248	0.548	0.591	
Full model		−4.709	22.682	−0.208	0.838	0.405
vSTM	Age	0.234	0.087	2.702	0.016	
	Naming (EOWPVT-R)	−0.111	0.188	−0.589	0.564	
	WWP index	16.369	42.051	0.389	0.702	
Full model		−7.719	38.092	−0.203	0.842	0.400

## Data Availability

The data that support the findings of this study are available on request from the corresponding author. The data are not publicly available due to privacy or ethical restrictions.

## Abbreviations

The following abbreviations are used in this manuscript:

IQ	Intelligence Quotient
ADHD	Attention Deficit Hyparctivity Disorder
ASD	Autism Spectrum Disorder
HF-ASD	High-functioning Autism
TD	Typically Developing
STM	Short-term memory
WM	Working Memory
vSTM	Verbal Short-term Memory
vWM	Verbal Working Memory
PIQ	Performance Intelligence Quotient
VIQ	Verbal Intelligence Quotient
CPM	Colored Progressive Matrices
CVS	Chrichton Vocabulary Scale
SCQ	Social Communication Questionnaire
EOWPVT-R	Expressive One-Word Picture Vocabulary Test-Revised
WWM	Whole-Word-Match
PCC	Percentage of Consonant Correct
V	Vowel
C	Consonant
SD	Standard Deviation
RS	Raw Score
